# Hierarchical Zeolites as Catalysts for Biodiesel Production from Waste Frying Oils to Overcome Mass Transfer Limitations

**DOI:** 10.3390/molecules26164879

**Published:** 2021-08-12

**Authors:** Elyssa G. Fawaz, Darine A. Salam, Severinne S. Rigolet, T. Jean Daou

**Affiliations:** 1 Department of Civil and Environmental Engineering, Maroun Semaan Faculty of Engineering and Architecture, American University of Beirut, Riad El Solh, Beirut P.O. Box 11-0236, Lebanon; egf00@aub.edu.lb; 2 Axe Matériaux à Porosité Contrôlée (MPC), Institut de Science des Matériaux de Mulhouse (IS2M), Université de Haute Alsace, UMR CNRS 7361, ENSCMu, 68093 Mulhouse, France; severinne.rigolet@uha.fr (S.S.R.); jean.daou@uha.fr (T.J.D.); 3 Université de Strasbourg, 67081 Strasbourg, France

**Keywords:** zeolites, hierarchical, transesterification, acidity, mass transfer limitation

## Abstract

Hierarchical crystals with short diffusion path, conventional microcrystals and nanocrystals of ZSM-5 zeolites were used for biodiesel production from waste frying oils and were assessed for their catalytic activity in regard to their pore structure and acidic properties. Produced zeolites were characterized using XRD, nitrogen adsorption–desorption, SEM, TEM, X-ray fluorescence, and FTIR. Pore size effect on molecular diffusion limitation was assessed by Thiele modulus calculations and turnover frequencies (TOF) were used to discuss the correlation between acidic character and catalytic performance of the zeolites. Owing to the enhanced accessibility and mass transfer of triglycerides and free fatty acids to the elemental active zeolitic structure, the catalytic performance of nanosponge and nanosheet hierarchical zeolites was the highest. A maximum yield of 48.29% was reached for the transesterification of waste frying oils (WFOs) using HZSM-5 nanosheets at 12:1 methanol to WFOs molar ratio, 180 °C, 10 wt % catalyst loading, and 4 h reaction time. Although HZSM-5 nanosponges achieved high conversions, these more hydrophilic zeolites did not function according to their entire acidic strength in comparison to HZSM-5 nanosheets. NSh-HZSM5 catalytic performance was still high after 4 consecutive cycles as a result of the zeolite regeneration.

## 1. Introduction

In recent years, non-renewable resources have been over-exploited which led to an increase in the prices of petroleum-based fuels and to the exhaustion of energy reserves [1,2]. A fitting and sustainable alternative for current fuels is thus needed. Interest in biodiesel has been rising in recent years because of its renewability, minor dependence on depleted resources, and its lower pollution profile [3,4]. However, its high cost in comparison with petroleum-based fuels is a main obstacle to its commercialization owing to the raw material used for its production [5]. Besides the economic appeal it provides, waste frying oil (WFO) is one of the most promising biodiesel feedstocks by way of decreasing the quantities of WFOs being discarded into sewers and landfills [6]. However, WFOs contain between 0.5 wt % and 15 wt % of free fatty acid (FFAs) compared to less than 0.5 wt % for refined vegetable oils [7]. Therefore, basic homogeneous transesterification reactions are not favored for the conversion of these inexpensive oils. The base-catalyzed transesterification holds severe limitations in terms of soap formation due the presence of high water contents and FFAs in the feedstock, in terms of the produced methyl esters separation from glycerol [8]. Homogeneous acid catalysts are less sensitive to FFAs content but require a long reaction time, a neutralization step and produce a mass of salt residues, which causes engine corrosion [9,10]. Acid heterogeneous catalysts offer a better alternative owing to their environmental friendliness, easy removal, and non-corrosive character [11,12]. Madhuvilakku and Piraman assessed the transesterification of palm oil using ZnO and TiO_2_-ZnO metal oxides nanocatalyst produced by urea using a glycerol-nitrate combustion method. TiO_2_-ZnO (0.91 wt %) showed the best catalytic activity achieving a conversion of 92.2% after 5 h reaction time at 60 °C and a 6:1 methanol to oil ratio [13]. Feyzi et al. evaluated CsH_2_PW12O 40Fe-SiO_2_ nano-catalyst for biodiesel production from sunflower oil. They obtained a yield of 81% with a methanol to oil ratio of 12:1, after 240 min of reaction at 60 °C [14]. Gardy et al. transesterified WFOs using TiO_2_/PrSO_3_H mesoporous acid catalyst (4.5 wt %) and obtained a yield of 98.3% at a methanol to oil ratio of 15:1, and a 60 °C [15]. Teo et al. synthesized CaSO_4_/Fe_2_O_3_-SiO_2_ catalyst, which owed to its crystal small size, large specific surface area and well-defined hierarchically porous structure, showed high reactivity (94%) in the transesterification of *Jatropha curca* oil under reaction conditions of 12 wt % catalyst loading and methanol to oil ratio of 9:1 at 120 °C for 4 h [16]. Kuniyil et al. achieved the highest catalytic activity (97.1%) towards the transesterification of WFO with the ZnCuO/(30%)NDG catalyst at the optimized conditions of 180 °C, 8 h, 10 wt % catalyst loading, and a methanol:oil molar ratio of 15:1 [17].

An effective type of heterogeneous solid acids, zeolites have high crystallinity, specific surface area, porosity, controllable acidity, and hydrophobicity. Different studies followed an ion exchange procedure to form and compare between microporous protonated zeolites, which is the most common form of solid acid heterogeneous zeolite catalysts [18,19,20,21]. However, physical properties of microporous acid zeolites limit bulky molecules such as triglycerides from entering the narrow pores and reaching the internal active sites necessary for their conversion to fatty acid methyl esters (FAMEs). Furthermore, molecules that are able to convert into more valuable products have difficulty escaping the zeolite through the narrow micropores, yielding low feedstock conversion. An effective way of developing improved zeolites activity for biodiesel production is the synthesis of materials with short diffusion paths and hierarchical porosity which overcome diffusional limitation by having secondary mesoporosity while maintaining the high reactivity of microporous acid zeolites as shown for similar and other catalytic and adsorption applications [22,23,24,25,26,27,28,29,30]. To our knowledge, no studies have yet assessed the transesterification of WFOs for the production of biodiesel using hierarchical zeolites. In the current study, the influence of pore structure and acidic properties of ZSM-5 zeolites displaying distinctive crystal morphologies (conventional microcrystals (coffin-shaped), nanocrystals, nanosponges, and nanosheets) has been evaluated for the transesterification of WFOs to biodiesel. The catalytic behavior of the ZSM-5 zeolites was optimized by varying the reaction temperature, the load of catalyst, the methanol to WFO molar ratio and the reaction time. Thiele modulus model and turnover frequencies (TOF) were used to discuss the correlation between pore size and acidic character of the catalysts, and their performance upon laboratory outcomes.

## 2. Experimental Section

### 2.1. Catalysts Synthesis

In a 45 mL stainless steel ^®^Teflon-lined autoclave (USA), sulfuric acid (Aldrich, St. Louis, MI, USA) and tetraethoxysilane (Aldrich, 98%) were dissolved in distilled water to produce the ZSM-5 nanosheets. Al_2_(SO_4_)_3_.18H_2_O (Rectapur, 99%, UK) and sodium hydroxide (Riedel de Haen, 99%, USA) were then supplemented. The structuring agent C22-6-6 (C_22_H_45_-N^+^(CH_3_)_2_-C_6_H_12_-N^+^(CH_3_)_2_-C_6_H_13_Br_2_) which was produced in two steps as per a modified method reported by Na et al. [31], was finally added to obtain the desired molar composition of the gel. The gel was then placed in a 30-rpm tumbling oven for 4 days at 150 °C, after having been stirred at 1000 rpm for 30 min, and heated at 60 °C for 4 h.

To produce ZSM-5 nanosponges, sodium hydroxide (Riedel de Haen, 99%), sodium aluminate (NaAlO_2_ of 56.7 wt % Al_2_O_3_, 39.5 wt % Na_2_O and 3.3 wt % H_2_O), EtOH (99%), tetraethoxysilane (TEOS, Aldrich, 98%), and 18–N_3_–18 (prepared through organic reactions as defined by Na et al. [32]) were stirred at 1000 rpm and dissolved in distilled water for 30 min at room temperature. The mixture was then heated for 6 h at 60 °C to finally be placed at 150 °C for 5 days, in a 30 rpm tumbling oven.

For comparison, ZSM-5 zeolite nanocrystals were produced by dissolving tetrapropylammonium hydroxide (25 wt % TPAOH-aqueous solution, Fluka, USA) and C_9_H_21_O_3_Al in distilled water. The mixture was stirred at 25 °C for 20 min before the addition of NaBr. The mixture was then further stirred for 20 min, and porous silica gel (100 mesh) was added. The gel was ultimately heated at 170 °C for 24 h.

Typical coffin-shaped ZSM-5 zeolite large crystals were produced under similar conditions as ZSM-5 nanosheets. The sole variation being the usage of a tetrapropylammonium hydroxide aqueous solution (25 wt %, Fluka) as an alternative to C22-6-6 [33].

The zeolites’ gel compositions are displayed in Section S1 of the Supplementary Materials.

After production, ZSM-5 zeolites were calcined in air at 550 °C for 8 h after being cleaned with distilled water and dried up at 105 °C for 12 h.

ZSM-5 zeolites were then exchanged with NH_4_^+^ ions by 1 M NH_4_Cl aqueous solution during 2 h at 80 °C. The reactions were performed under stirring, in round bottom flasks fitted with reflux condensers. The process of ion exchange was replicated three times at a zeolite mass (g) to solution volume (mL) ratio of 1:20. NH_4_^+^-ion-exchanged zeolites were repetitively washed with distilled water. After filtration and drying at 105 °C for 12 h, H^+^ form zeolites were obtained by calcining NH_4_^+^-ion-exchanged zeolites in air at 550 °C for 10 h. The different exchanged HZSM-5 microcrystals, nanocrystals, nanosheet, and nanosponges were given respective labels MC-HZSM5, NC-HZSM5, NSh-HZSM5, and NS-HZSM5. Residual Na content was checked via X-ray fluorescence and its content was negligeable throughout all samples.

The characterization of the different catalysts is described in detail in Section S2 of the Supplementary Materials.

### 2.2. Esterification Reaction Procedure

For all HZSM-5 synthesized, the transesterification reaction of WFOs with methanol (Chromasolv, 99.9%) was performed in triplicates under stirring in a 50 mL reactor, fitted with a reflux condenser. In the reactor, 0.6 mL of WFO were loaded. Methanol was added in the desired amount to achieve alcohol/oil molar ratios of 6:1, 12:1, or 25:1. The loading of the catalyst was also varied at 5, 7.5, or 10 wt % with respect to the WFO mass. The catalyst was dried at 105 °C prior to reaction and the reactors contents were heated at 140 °C for 6 h. When the reactions were completed, the produced fatty acid methyl esters (FAMEs) and remaining FFAs and triglycerides (TGCs) were extracted for analysis. The reactors were first extracted with 50 mL methanol, then by two 50 mL-DCM (dichloromethane) (Fisher, 99.8%) extraction, each. Liquid extracts and catalysts were then split through a bed of beads and a 0.2 µm filter paper, by gravity filtration. Transesterification reaction temperature and time were later optimized for the HZSM-5 type that indicated the highest oil conversion. Tested reaction temperatures were 60, 140, and 180 °C. The temperature which yielded the highest oil conversion was then adopted in to define the optimum reaction time. For this aim, a kinetic study was conducted with reaction times varying between 0 and 24 h (0, 0.5, 1, 2, 3, 4, 6, 8, 16, 24). At each defined reaction time, triplicate samples were sacrificed, and FAMEs yield was measured. Blanks were also performed in triplicates for each parameter variation.

### 2.3. Chemical Analysis

The content of FAMEs in the liquid extracts was quantified by gas chromatography (Trace GC Ultra, Thermo Scientific, Waltham, MA, USA) according to a method described by Fawaz et al. [34]. Reference standard (Supelco 37 component FAME mix in DCM, TraceCert) was used to identify peaks of methyl esters. The biodiesel yield was defined by Equation (1)

(1) $$Yield\;\left(\%\right)=\frac{\sum C_{FAMEs}\times V_{extract}}{M_{0\;\;WFO}}\times 100$$

where, $C_{FAMEs}$ is the concentration of FAMES in mg/L, $V_{extract}$ is the liquid extract volume (L), and $M_{0\;WFO}$, the WFO initial mass that was added to the reactor (mg).

Residual FFAs and triglycerides were quantified using reversed-phase HPLC using two different procedures, as per Gratzfeld-HüSgen and Schuster [35]. For the two methods, a Chromatographic System equipped with a diode array detector (Agilent Technologies, Santa Clara, CA, USA) was used and a C_8_ (150 × 2.1 mm ZORBAX Eclipse XDB, 5 μm) column was adopted.

FFAs residual content was determined using a detection wavelength of 258 nm. Bromophenacyl bromide (Fluka) was used to derivatize FFAs prior to sample injection to obtain their corresponding esters. A mobile phase consisting of (A) water and acetonirile + (B) 1% tetrahydrofuran was used. The solvent gradient used for this method was as follow: 0 min 30% B; 15 min 70% B; 25 min 98% B.

For triglycerides determination, a mix of (A) water and (B) acetonitrile/methyl-*t*-butyl-ether (9:1) constituted the mobile phase. Solvent gradient covered 87% B at 0 min; and 100% B at 25 min. The products were detected at 215 nm.

The kinetics of the transesterification reactions are described in detail in Section S3 of the Supplementary Materials.

## 3. Results and Discussion

### 3.1. Characterization of the Catalysts

#### 3.1.1. XRD

**Figure 1:**
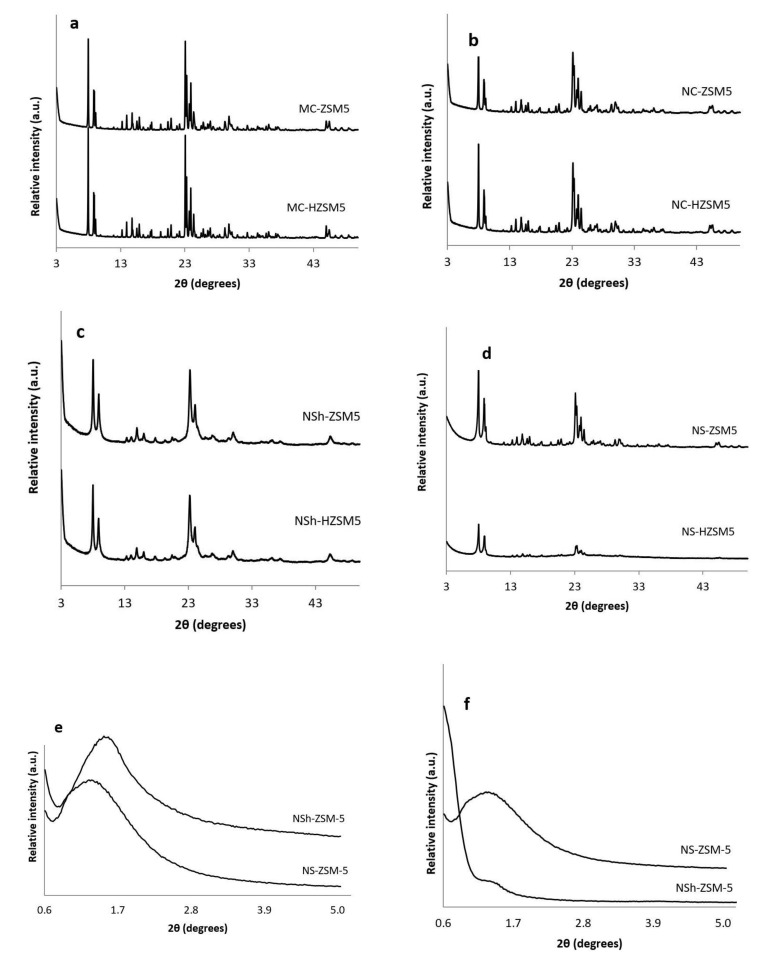
XRD diffractograms of (**a**) calcined MC-ZSM5 and MC-HZSM5 at wide angles, (**b**) calcined NC-ZSM5 and NC-HZSM5 at wide angle patterns, (**c**) calcined NSh-ZSM5 and NSh-HZSM5 at wide-angles, (**d**) calcined NS-ZSM5 and NS-HZSM5 at wide-angles, (**e**) non-calcined NSh-ZSM5 and NS-ZSM5 at low-angles, (**f**) calcined NSh-ZSM5 and NS-ZSM5 at low-angles.

Purity and crystallinity of the different morphologies of ZSM-5 and H-ZSM-5 zeolites were assessed by XRD (Figure 1). As illustrated in Figure 1a–d the different synthesized zeolites were pure MFI crystalline phase. In Figure 1a,b, NC-HZSM5 and MC-HZSM5 samples were well crystallized. However, XRD reflections of NC-HZSM5 showed wider peaks than the peaks of MC-HZSM5 which suggests the presence of smaller crystal sizes. After exchange with NH_4_^+^, the hierarchical zeolites; NSh-HZSM5 and NS-HZSM5 presented XRD peaks with broader peaks, which suggests the presence of even reduced domains of crystals, further confirmed through imaging (Figure 1b,d). Also, repeated ion exchange treatment conditions might have affected the relative crystallinity of NS-HZSM5 [36]. At wide angles, the main portion of diffraction peaks that can be accurately indexed, belonged to the h0l crystallographic plane for NSh-HZSM5. This suggests that the crystal growth alongside the b-crystal axis is inhibited by the hydrophobic tail of structure directing agent, confirming thus the formation of nanosheets [31]. A 2D hexagonal symmetry of micropores piled in two distinct orientations is suggested for NS-HZSM-5 as mentioned by Na et al. [32]. Adopting C22-6-6 and 18-N_3_-18 as structure directing agents in the production of nanosheets and nanosponges respectively, created meso-structures further identified by the existence of wide peaks at low diffraction angles (0.6° < 2ϴ < 4.7°) as shown in Figure 1e,f. These peaks disappeared after calcination for the nanosheets and were maintained intact for nanosponges [24]. This indicates that nanosponges samples should have higher mesoporous volume (further confirmed by N_2_ sorption tests). Relative crystallinities of NC-HZSM5, NSh-HZSM5, and NS-HZSM5 were 96.6%, 99.8%, and 78.4% respectively, compared to MC-HZSM5.

#### 3.1.2. SEM and TEM

**Figure 2:**
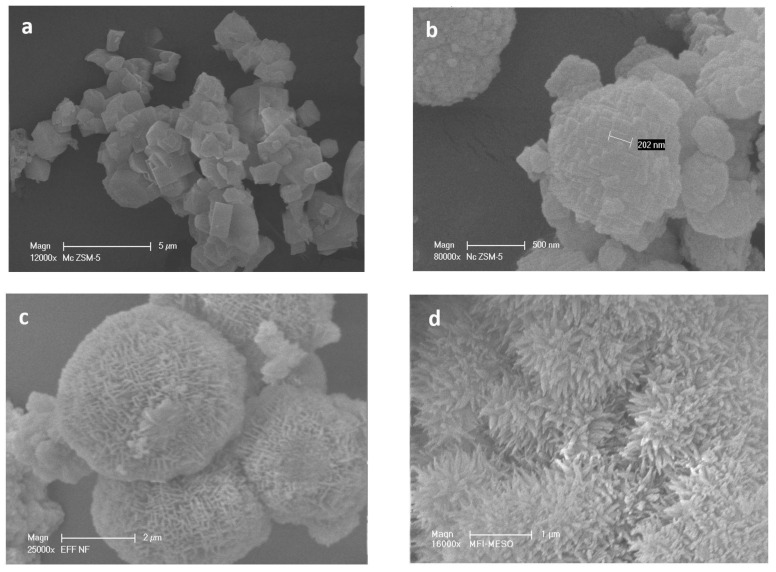
Scanning electronic microscopy (SEM) images of (**a**) MC-HZSM5, (**b**) NC-HZSM5, (**c**) NSh-HZSM5, and (**d**) NS-HZSM5.

**Figure 3:**
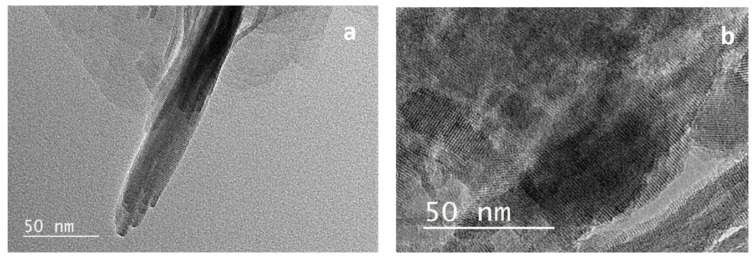
Transmission electronic microscopy (TEM) images of (**a**) NSh-HZSM5 and (**b**) NS-HZSM5.

The different morphologies of the HZSM-5 zeolite samples were observed by SEM images (Figure 2). MC-HZSM5 showed crystal sizes of 3.2 and 7.3 μm (Figure 2a). NC-HZSM5 revealed agglomerated crystals with a particle size ranging between 80 and 330 nm (Figure 2b). the production of lamellar materials of NSh-HZSM5 was a result of the C22-6-6 structure directing agent usage. The thickness of the lamellar stacking of nanosheets ranged between 20 and 40 nm composed of 2 nm in thickness zeolite nanosheets (Figure 2c and Figure 3a).

When 18-N_3_-18 was used as structure directing agent, the increase in the quaternary ammonium number and in carbon chain length produced the morphology of the NS-HZSM5 nanosponges (Figure 2d). The nanomaterials show to be spiky-like and uniform in shape with a size of 250–350 nm. TEM images of NS-HZSM5 (Figure 3b) showed interconnected walls of mesopore.

#### 3.1.3. Nitrogen Adsorption–Desorption

**Figure 4:**
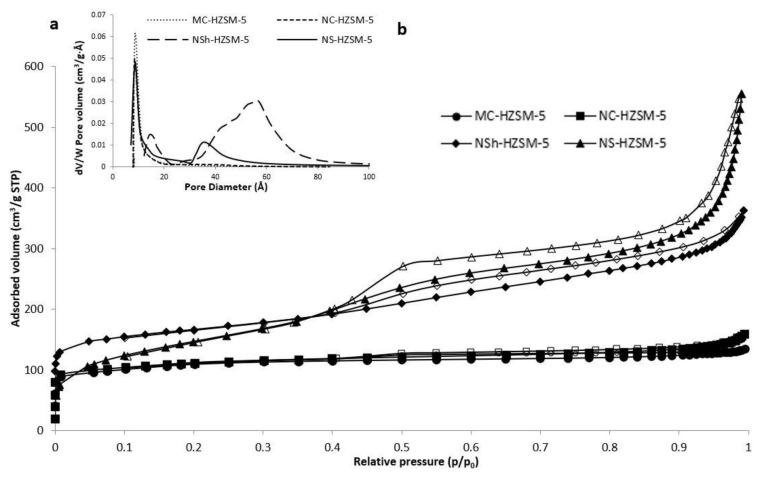
(**a**)Adsorption/desorption isotherms of nitrogen on the calcined MC-HZSM5, NC-HZSM5, NSh-HZSM5, and NS-HZSM5 samples, at −196 °C, (**b**) Micropore and mesopore size distribution of the exchanged and calcined zeolites.

Adsorption–desorption isotherms of nitrogen of the different calcined samples of HZSM-5 are shown in Figure 4a.

As expected for microporous solids, MC-HZSM5 and NC-HZSM5 displayed both isotherms of type I. Micropore volumes of MC-HZSM5 and NC-HZSM5 were 0.17 cm^3^·g^−1^ for both zeolites (Table 1). However, due to nanocrystals agglomeration (interparticular N_2_ adsorption), a small hysteresis was detected for NC-HZSM5. At low *p*/*p*_0_, the adsorption isotherm of NSh-HZSM5 is of type I and at high *p*/*p*_0_, it is of type II with H4 hysteresis [37]. The occurrence of a hysteresis loop at 0.4 < *p*/*p*_0_ < 1 is characteristic of a material with lamellar structures, as a result of nanosheets stacking. At low *p*/*p*_0_, NS-HZSM5 show type Ib isotherms, with a mesoporous presence indicated by the type II and type IV isotherms at 0.4 < *p*/*p*_0_ < 0.6. An interparticle adsorption of N_2_ is observed at *p*/*p*_0_ > 0.9. The mesoporous volume of NS-HZSM5 is assessed at 0.35 cm^3^·g^−1^. The BJH pore size distribution of NS-HZSM5 was intense and broad showing an average mesoporous diameter of 58 Å (Figure 4b). However, for NSh-HZSM5 zeolites, it was weaker and narrow, displaying a mean mesoporous diameter of 35 Å. Both hierarchical zeolites preserved the microporosity which clearly confirms the existence of mesopores and micropores at the same time in the samples. This is in accordance with the characterization results of XRD, TEM, and SEM. However, the micropore volume of 0.27 cm^3^·g^−1^ (Table 1) determined for the NS-HZSM5 from the t-plot technique is higher than those of other zeolites and larger than the ones anticipated for an MFI-type zeolite. The nanosponge-like zeolite relatively higher microporous volume in comparison with the other HZSM-5 morphologies is justified by the presence of a secondary microporosity caused by 18-N_3_-18, along with the MFI-type lattice prompted microporosity [32]. The BET surface and total pore volume presented in Table 1 are greater for the hierarchical zeolites relatively to the microporous ones. BET surface area increased as shorter diffusional paths (nanocrystals) and hierarchical porosity were created. (395 m^2^·g^−1^ was found for microporous zeolites, 411 m^2^·g^−1^ for nanometric NC-HZSM5 and 521 m^2^·g^−1^and 613 m^2^·g^−1^ for hierarchical nanosheets and nanosponges, respectively). The same pattern was observed with BEA-type zeolites synthesized by Astafan et al. [22]. The S BET increased from 626 m^2^·g^−1^ for micron-sized crystals to 726 m^2^·g^−1^ for nanometer sized zeolites and 977 m^2^·g^−1^ for nanosponge BEA-type crystals, respectively.

#### 3.1.4. FTIR and ^27^Al MAS NMR

For zeolite acidity characterization, FTIR was used. The bands produced by the adsorption of basic pyridine probe molecules were measured. Pyridine helps identify and quantify the acidity of the active sites and distinguishes between the O-H acidic groups, which represent the Brønsted acid centers by forming pyridium ions on protonic sites (PyrH^+^), and the weaker Lewis acid groups to which pyridine is physically bonded (PyrL). Also, the probe molecule has a diameter of 5.4 Å [38] which makes possible its ease of access to the inner porosity of the HZSM-5 zeolites of 5.3–5.6 Å pore diameter.

**Figure 5:**
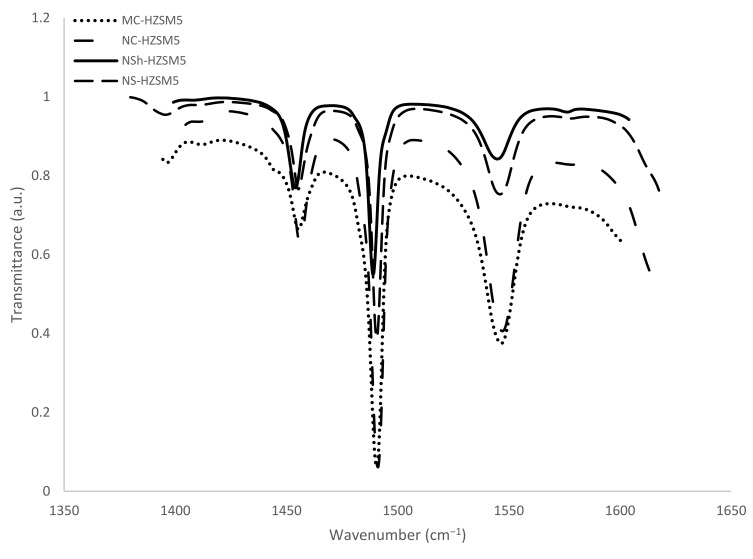
FTIR spectra upon the saturation of zeolites with pyridine, followed by the desorption at 150 °C.

Bands absorbance areas at 1545 and 1454 cm^−1^ were detected to calculate the Brønsted and Lewis concentration centers apt to react with pyridine at 150 °C (Figure 5). Previously defined extinction coefficients (1.28 and 1.13 cm·mol^−1^ for pyridine retained respectively on Lewis and Brønsted sites [39]) were used for the concentrations calculation. Pyridine reacting with both Brønsted and Lewis sites is observed at 1490 cm^−1^ but does not allow the differentiation between the two acidic sites. It was not therefore applied for acidity characterization. The results are presented in Table 2. Brønsted acidity decreased for the hierarchical zeolites relatively to the microporous ones, while Lewis acidity increased. Better catalytic performance is hence anticipated for the more acidic MC-HZSM5 and NC-HZSM5 zeolites. Miranda et al. [26] also assessed the Lewis and Bronsted sites concentrations that are able to interact with pyridine at 150 °C for micron-sized, nano-sized, nanosheets, and nanosponges-type MFI zeolites and obtained comparable concentrations for PyrH^+^ (304 µmol·g^−1^, 332 µmol·g^−1^, 83 µmol·g^−1^, and 151 µmol·g^−1^, respectively) and for PyrL (44 µmol·g^−1^, 25 µmol·g^−1^, 119 µmol·g^−1^, and 44 µmol·g^−1^, respectively).

^27^Al solid state MAS NMR spectra for MC-HZSM5, NC-HZSM5, NSh-HZSM5, and NS-HZSM5 zeolite samples were recorded and explained (Section S4 in Supplementary Materials). Correspondingly, the different zeolites’ Si/Al framework ratios were determined (Table 2).

### 3.2. Optimized Reaction Conditions of Transesterification

#### 3.2.1. Catalyst Loading Effect

**Figure 6:**
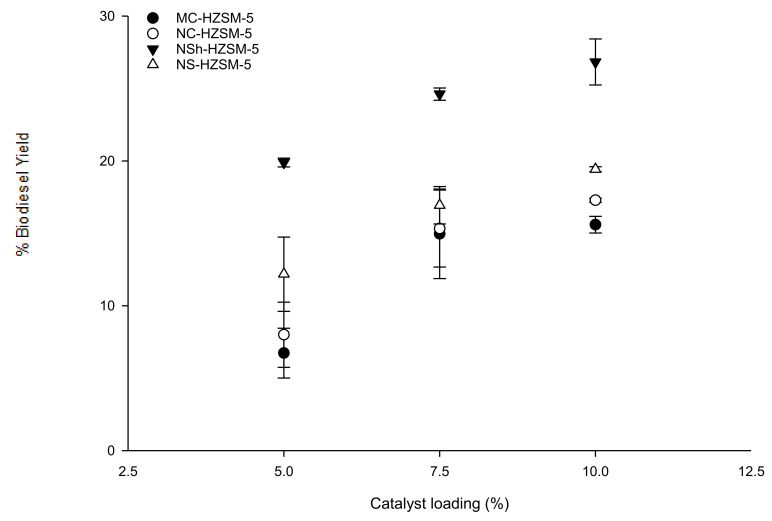
Effect of MC-HZSM5, NC-HZSM5, NSh-HZSM5, and NS-HZSM5 catalysts loading on the transesterification of WFO at the molar ratio of methanol to WFO of 12:1, 140 °C, 550 rpm, and 6 h.

Catalyst loading effect on triglycerides conversion was evaluated for MC-HZSM5, NC-HZSM5, NSh-HZSM5, and NS-HZSM5. Experiments were performed with loadings of 5, 7.5, and 10 wt % with respect to the used mass of WFO, at similar reaction conditions of 140 °C reaction temperature, 12:1 methanol to WFOs molar ratio, 550 rpm stirring rate, and 6 h reaction time. For all zeolite catalysts, FAMEs yield increased with increasing catalyst loading with an conversion order of MC-HZSM5 (15.60%, SD = 0.57) < NC-HZSM5 (17.29%, SD = 0.14) < NS-HZSM5 (19.43%, SD = 0.18) < NSh-HZSM5 (26.83%, SD = 1.59) (Figure 6). At increased catalyst loadings, higher amounts of acid active sites will be accessible for the oil to react and produce FAMEs [40]. In fact, the oil solubility in methanol is limited which leads to the formation of three phases in the heterogeneous transesterification (methanol, WFO, and solid zeolite). The transesterification can thus only happen at the interface of the methanol and WFO phases. Increasing catalysts loading will enhance the concentration of active sites at the interface between the methanol and oil which will enhance the formation of FAMEs and hence, higher conversions of oil will be achieved [41].

#### 3.2.2. Methanol to WFO Ratio Effect on Transesterification

**Figure 7:**
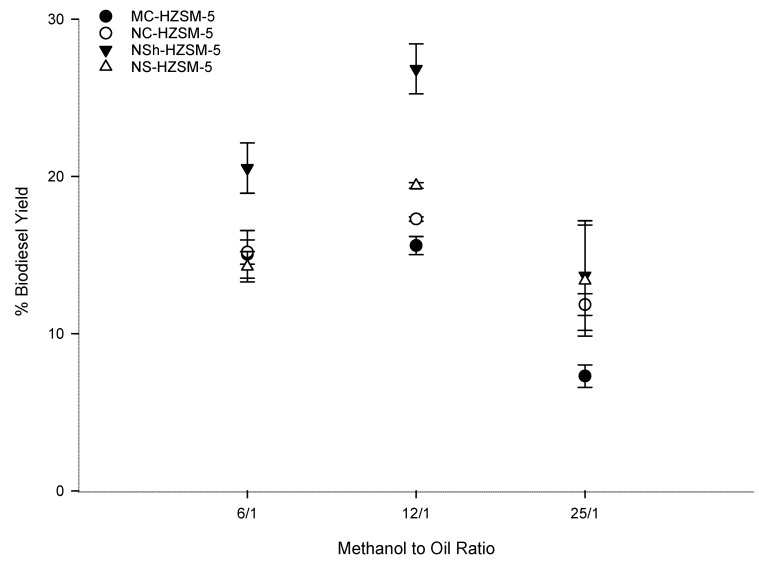
Effect of methanol to WFO molar ration variation on the transesterification of WFO using MC-HZSM5, NC-HZSM5, NSh-HZSM5, and NS-HZSM5 catalysts, at 10 wt % catalyst loading, 140 °C, 550 rpm, and 6 h.

Being a reversible reaction, excess methanol should be used to direct the transesterification to FAMEs production [42]. Increased methanol amount effect on triglycerides conversion was assessed by varying the molar ratio of methanol to WFOs (6:1, 12:1, 25:1) for the different catalysts. Fixed optimal reaction conditions consisting of 6 h reaction time, 10 wt % catalyst loading, 550 rpm stirring rate, and 140 °C reaction temperature were used. Figure 7 shows that as the molar ratio of methanol to WFO increased from 6:1 to 12:1, the conversion rate of WFO increased for the different zeolites. This increase was moderate for the microcrystals and more pronounced for the hierarchical zeolites. Thus, excess methanol was demonstrated advantageous for triglycerides conversion into FAMEs. However, for all catalysts, FAMEs yield decreased as the methanol to WFO molar ratio was further increased to 25:1. This trend is owing to the minor recombination of FAMEs and glycerol which produce monoglycerides. Excess methanol is helpful for the conversion of triglycerides into monoglycerides. Nevertheless, monoglycerides alter the solubility of the practically immiscible glycerol and FAMEs urging thus the backward reaction to happen [41,43]. 12:1 methanol to WFOs molar ratio was consequently the optimal molar ratio used for the rest of the optimization tests, with a conversion order that was kept at MC-HZSM5 (15.60%, SD = 0.57) < NC-HZSM5 (17.29%, SD = 0.14) < NS-HZSM5 (19.43%, SD = 0.18) < NSh-HZSM5 (26.83%, SD = 1.59).

#### 3.2.3. Reaction Temperature Effect on Transesterification

**Figure 8:**
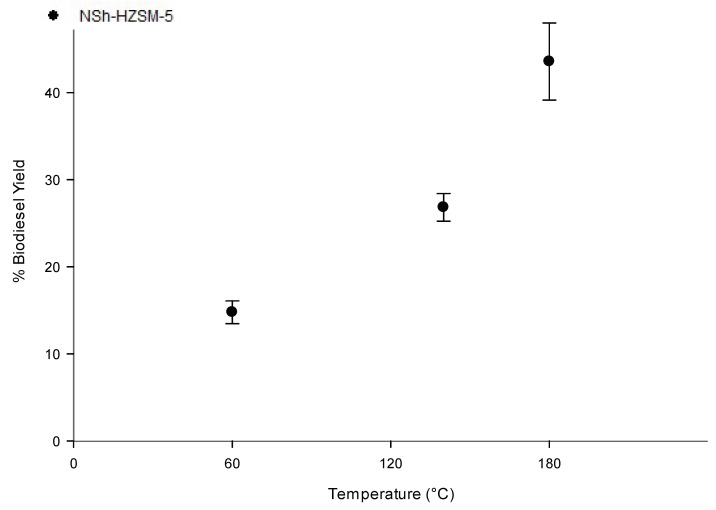
Effect of reaction temperature variation on the transesterification of WFO using NSh-HZSM5 catalyst at 10 wt % catalyst loading, methanol to WFO molar ratio of 12:1, 550 rpm, and 6 h.

To assess the reaction temperature effect on WFOs conversion, experiments employing the hierarchical NSh-HZSM5 catalyst, that reached the maximum conversion rates of WFO in the previous experiments, were carried out at three distinct temperatures of 60, 140, and 180 °C, under similar reaction conditions (optimal methanol to WFOs molar ratio = 12/1, optimal catalyst loading = 10%, stirring rate = 550 rpm, and reaction time = 6 h). As indicated in Figure 8, catalytic activity of NSh-HZSM5 increased with increasing temperature with the highest FAMEs yield of 43.57% (SD = 4.44) registered for reaction temperature of 180 °C. Higher conversions were achieved at higher temperatures because WFO viscosity decreased and hence promoted an improved phase mixture of methanol and WFOs [44]. With the increasing temperature, the average kinetic energy of the molecules triggers faster movement and more frequents collision which increases the rate of diffusion of molecules between the liquid phases and between the liquid phases and the catalyst’s solid phase [45]. Consequently, and corresponding to the Arrhenius equation [46], higher conversions are reached with higher temperatures.

#### 3.2.4. Reaction Time Effect on Transesterification

**Figure 9:**
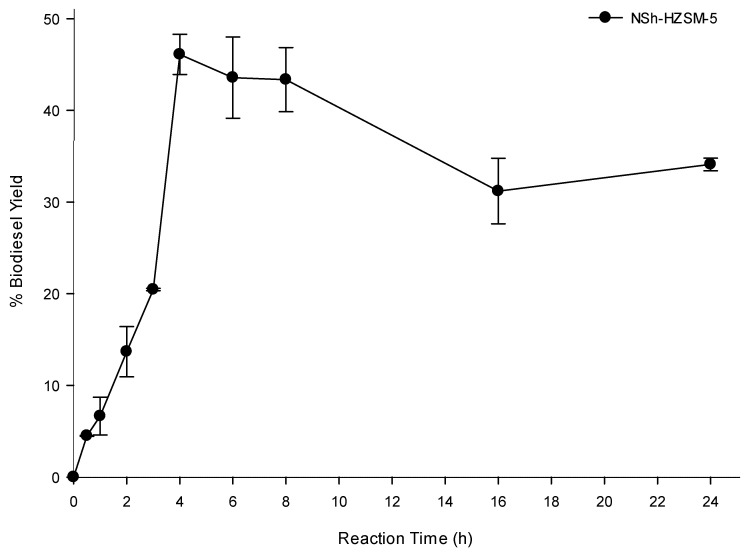
Effect of reaction time on the transesterification of WFO using NSh-HZSM5 catalyst at of 10 wt % catalyst loading, methanol to WFOs molar ratio of 12:1,180 °C, and 550 rpm.

To determine the optimal transesterification time which would lead to the highest oil conversion, experiments were carried out using hierarchical NSh-HZSM5 at the optimal reaction conditions of 12:1 methanol to WFO molar ratio, 180 °C reaction temperature, catalyst loading of 10%, and 550 rpm stirring rate, by varying the reaction time between 0 and 24 h. Figure 9 shows biodiesel yield as a function of reaction time.

A rise in the reaction time from 30 min to 2 h almost doubled the biodiesel yield which increased from 4.50% (SD = 0.04) to 13.68% (SD = 2.74). Additional increase in the reaction time caused a substantial increase in FAMEs yield reaching a maximum of 48.29% after 4 h. No significant enhancement was observed in WFO conversions beyond 4 h and up to 8 h of reaction time, after which a reduction in FAMEs yield was observed at 16 and 24 h reaction time. The latter decrease in the oil conversion is most probably due to the oil polymerization at prolonged heating time. Indeed, a solid mass was observed at the bottom of the reactors after 16 h and remained insoluble in DCM used for the oil extraction. Apart from generating smaller triglycerides conversions, higher reaction times are not advantageous because of the higher energy intake they need.

Section S5 in Supplementary Materials shows numeric FAMEs yield and remaining triglycerides and FFAs contents at the various reaction conditions.

#### 3.2.5. Mass Transfer Assessment of the Synthesized Catalysts on the Esterification Reaction

Results from the experiments showed that the acid strength does not appear to be the only reason influencing the conversions achieved. Therefore, the effect of the pore sizes on the internal mass transfer limitation was assessed.

The more the zeolites have ordered microporosity, the more they are acidic. However, the existence of this one type of microporosity seems to contribute to the small use of the zeolite active volume by imposing limitations on the accessibility and the molecular transport of big molecules such as triglycerides into the internal crystalline active acid sites [47]. Actually, microporous HZSM5 have a small pore size (5.1–5.5 Å) and triglycerides, diglycerides, and monoglycerides molecules possess kinetic diameters of around 11.81, 11.46, and 8.77 Å estimated respectively, using Equation (2) [48].

(2) $$\sigma =1.234\cdot {\left(M_{W}\right)}^{1/3}$$

where σ is the kinetic diameter anticipated for hydrocarbons molecule, and $M_{W\;}$ is the molecular weight of WFOs (876.5 g mol^−1^ for triglycerides (estimated using the method described by Sanchez et al., [49], 625 g mol^−1^ for diglycerides and 358.6 g mol^−1^ for monoglycerides [50]). This implies that the transport of oil molecules in pores of microporous zeolites such as HZSM-5 is hindered.

Two strategies were adopted and evaluated to enhance molecular mobility and accessibility to the active sites confined in zeolites for WFO transesterification. First, the microporous zeolite diffusion pathway length was reduced (NC-HZSM5) and second, a secondary mesoporosity was introduced within the microporous crystals (NSh-HZSM5 and NS-HZSM5).

To evaluate more precisely the magnitude of internal mass transfer limitations of the different zeolites on triglycerides conversions, reaction rates on the different morphologies of HZSM-5 were studied as a function of intrinsic diffusion rate by the Thiele modulus (∅) estimation (Equation (3)) [51].

(3) $$\emptyset =L\cdot \sqrt{\frac{k}{D_{eff}}}$$

where, L (m) denotes the characteristic diffusion length of the zeolite determined by SEM/2 [52], k (s^−1^) is the reaction rate coefficient, and $D_{eff}\;$(m^2^·s^−1^) the effective diffusivity of triglyceride and methanol inside the zeolitic pores.

This method therefore couples the effective diffusion coefficient theoretical estimate with the pseudo first-order rate constant k experimental value. A ∅ value < 0.1 designates that the transesterification reaction is not limited in terms of mass transfer and that observed reaction rate corresponds to the intrinsic one [53].

The effective diffusion coefficient $D_{eff}\;$ was found using Equation (4) [54].

(4) $$D_{eff}=D_{AB}\cdot \;\frac{\varepsilon }{\tau }$$

where $D_{AB}\;$(m^2^·s^−1^) is the molecular diffusion coefficient of WFO in methanol, τ is the zeolite pore tortuosity, and ε is the catalyst’s particle porosity.

Molecular diffusion coefficient of triglycerides in methanol was calculated to 1.57 × 10^−6^ m^2^·s^−1^ at 180 °C (Section S6 in Supplementary Materials), according to the Wilke-Chang method [55]. τ for all zeolites was given a value of 4 [51] and the different values of ε were determined by the pore fractions’ nitrogen adsorbing capacity.

Reaction rate constants of 0.075 m^−2^·h^−1^, 0.082 m^−2^·h^−1^, 0.125 m^−2^·h^−1^, 0.097 m^−2^·h^−1^ were calculated for MC-HZSM5, NC-HZSM5, NSh-HZSM5, and NS-HZSM5, respectively (Section S7 in Supplementary Materials). Corresponding $D_{eff}$ values appear hence in Table 3 with the Thiele modulus estimates.

Thiele modulus values of 0.0050, 0.0004, and 0.0033 were obtained for NC-HZSM5, NS-HZSM5, and NSh-HZSM5 respectively, whereas a value of ~0.2 was estimated for MC-HZSM5 which showed the lowest catalytic performance at different catalysts loading and methanol to WFO molar ratios, relatively to the other nanosized and hierarchical zeolites. This can be attributed to their poor diffusion properties since their active capacity was not entirely accessible to the triglycerides molecules despite the high Brønsted acid concentration MC-HZSM5 microcrystals hold. NC-HZSM5, NSh-HZSM5, and NS-HZSM5 displayed values of Thiele modulus <0.1. This validates that transport of molecules was improved by reducing diffusion length (NC-HZSM5) and generating an additional (meso)porosity (NSh-HZSM5 and NS-HZSM5). However, enhancing catalyst efficiency by shortening its diffusion length is not enough to accomplish a noteworthy increase in oil conversions. This is supported by the catalytic performance over NC-HZSM5 at the various catalysts loading and methanol to WFO molar ratios. the catalytic performance of these zeolites was lower than that of the hierarchical catalysts. Aside from their shorter diffusion length, producing (meso)pores which are reachable to the triglycerides and FFAs from the zeolite’s exterior, better improved the transport of the molecules towards and out of the acid sites of the hierarchical zeolites (NSh-HZSM5 and NS-HZSM5) which displayed the highest FAMEs yields at optimal transesterification conditions.

Nonetheless, there is evidence that amid the hierarchical zeolites, NS-HZSM5 crystals with a greater amount of Brønsted sites (218 v/s 103 µmol·g^−1^ for NSh-HZSM5) along with a bigger mesoporous volume and diameter (0.35 cm^3^·g^−1^ and 58 Å respectively versus 0.13 cm^3^·g^−1^ and 35 Å for NSh-HZSM5), exhibit lower catalytic activity than NSh-HZSM5 (19.43% (SD = 0.18) for NS-HZSM5 versus 26.83% (SD = 1.59) for NSh-HZSM-5) at the following transesterification conditions (10 wt % catalyst loading, 550 rpm stirring rate, 140 °C reaction temperature, and 6 h reaction time).

To give understanding into which major aspects affect the catalytic performance, turnover frequencies (TOF) were thus estimated for the hierarchical zeolites. 1.12 × 10^−5^ and 1.21 × 10^−5^ mol_produced FAMES_ .µmol_H_^+^·m^−2^·g·h^−1^ for NSh-HZSM5 and NS-HZSM5, were the respective calculated TOF values respectively. These values are very comparable though their reactivity was significantly different. This indicates that there is no considerable impact of the Brønsted concentrations on the catalytic performance of NSh-HZSM5 and NS-HZSM5.

Generally, in addition to the number of porosity levels, hierarchical zeolites are characterized by their individual geometry. NS-HZSM5 nanosponges have a disordered geometry in comparison with the very ordered NSh-HZSM5 nanosheets geometry [31,32]. The primary purpose of using hierarchical zeolites as catalysts is to couple the enhanced molecular transport and the catalytic character of micropores by introducing additional mesopores, in a single material. However, the connectivity amid the micropores and the mesopores is crucial to improve the effectiveness of bi-porosity in catalyzed reaction [56] and consequently, facilitate the transport of bulky materials from the external mesoporous surface of the hierarchical zeolites into the active intercrystalline space. Although NS-HZSM5 zeolites have higher concentrations of Brønsted sites, surfactant masses could have engendered non-orderly (meso)pores that were ineffective for the selective transport of triglycerides and FFAs to the entirety of the intrinsic acid sites, relatively to possibly more arranged (meso)pores in NSh-HZSM5. Consequently, a more precise modeling of mesopores located in harmony with the micropores is required to engineer hierarchical zeolites for high catalytic selectivity. Nevertheless, similar causations are hard to give with cert with no additional evaluation. Thus, the matter will be disregarded in this work.

Alternatively, a competing adsorption phenomenon might have existed between all reaction components on the surface of NS-HZSM5 zeolite. The relatively more hydrophilic NS-HZSM5 (Si/Al = 33, versus Si/Al = 55 for NSh-HZSM5) adsorbed glycerol by-product more strongly than less polar reactants and consequently blocked the passage of methanol, triglycerides, and FFAs to the acid sites of the zeolite [57]. However, when Si/Al ratio is high, as in the case of NSh-HZSM5, glycerol produced during the transesterification reaction desorbs quickly from the more hydrophobic zeolites’ surface, increasing thus the outer mesoporous surface coverage of methanol, triglycerides, and FFAs and their diffusion into the internal active sites. In terms of diffusion limitation, relative hydrophobicity, and reactant selectivity, NSh-HZSM5 showed the optimal catalytic performance in transesterification reactions for the production of biodiesel.

#### 3.2.6. Catalyst Regeneration

**Figure 10:**
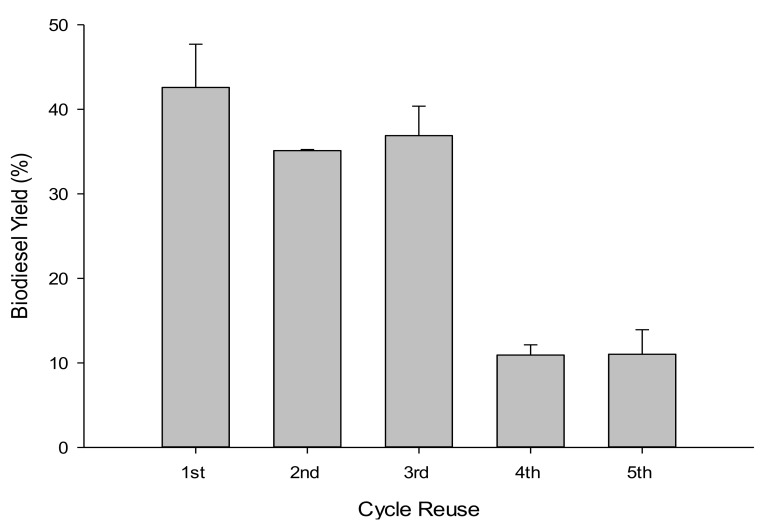
Biodiesel yield assessment in WFOs transesterification after NSh-HZSM-5 reuse.

Five successive 4 h WFOs transesterification cycles were performed using 10 g of WFOs at the optimal determined conditions (catalyst loading of 10 wt %, methanol to WFOs molar ratio of 12:1, reaction temperature of 180 °C, reaction time of 4 h, and stirring rate of 550 rpm), to assess the reusability of NSh-HZSM5 catalysts. At every cycle’s end, the catalysts were removed from the liquid products by filtration and dried at 105 °C for 6 h. A maximum yield of 47.71% was obtained for the first cycle and decreased to around 36% during the second and third cycles (Figure 10). At the end of the fourth and fifth cycles, only 11% biodiesel was obtained. This decrease indicates that NSh-HZSM5 does not retain catalytic activity after three successive cycles. This deactivation is possibly consequent to catalyst poisoning by impurities that constitute the WFOs aside from FFAs and triglycerides. WFOs are in fact subject to autoxidation giving rise to supplementary compounds like peroxides, aldehydes, and ketones. These could compete with triglycerides and FFAs molecules for directly accessing the mesopores of the hierarchical zeolite to the intrinsic active sites, hindering, and possibly obstructing the conversion of the desired reactants to FAMEs. The decrease in catalytic activity of the zeolite might also be due to glycerol by-product adsorption to the surface of the catalyst at each cycle, preventing thus reactants mass transfer to the acid sites. Selectivity of the acid catalyst towards all molecules present in the waste oil should thus be further investigated.

## 4. Conclusions

Despite their higher acidic strength, traditional large and nanocrystals of HZSM-5 zeolites achieved lower yields than nanosponge and nanosheet HZSM-5 zeolites, for the transesterification of WFO with methanol. Enhanced accessibility and molecular transfer of triglycerides and FFAs from the external mesoporous area to the effective intra-crystalline zeolite produced higher conversions of 19.43% (SD = 0.18) for NS-HZSM5 and 26.83% (SD = 1.59) for NSh-HZSM5 at identical transesterification conditions (catalyst loading of 10 wt %, stirring rate of 550 rpm, reaction temperature of 140 °C, methanol to WFOs ration of 12:1, and reaction time of 6 h). A maximum FAMEs yield of 48.29% was achieved for the transesterification of WFO with NSh-HZSM5 catalyst at 10 wt % catalyst loading, reaction temperature of 180 °C, methanol to linoleic acid molar ratio of 12:1, 4 h reaction time, and was preserved for three successive cycles after catalyst regeneration. Despite having high acidity and accessibility, HZSM-5 nanosponges did not function to their complete capacity in comparison with HZSM-5 nanosheets, as their relatively lower Si/Al framework ratio makes their surface more prone to adsorb glycerol by-product than the less polar reactants.

## Figures and Tables

**Table 1:** Characteristics of the different HZSM-5 morphologies.

Type of HZSM-5	Si/Al Ratio	S BET (m^2^/g)	S External (m^2^·g^−1^)	S Micropore (m^2^·g^−^^1^)	Total Porous Volume (cm^3^/g)	Microporous Volume (cm^3^/g)	Mesoporous Volume (cm^3^/g)
MC-HZSM5	45	395	22	374	0.17	0.17	-
NC-HZSM5	25	411	34	377	0.17	0.17	-
NSh-HZSM5	44	521	175	346	0.32	0.19	0.13
NS-HZSM5	23	613	205	398	0.62	0.27	0.35

**Table 2:** Brønsted and Lewis acidity of the produced HZSM-5 catalysts.

Type of HZSM5	Si/Al Ratio ^a^	Si/Al of the Framework ^b^	[PyrH^+^] ^c^ (µmol·g^−1^)	[PyrL] ^d^ (µmol·g^−1^)
MC-HZSM5	45	53	351	44
NC-HZSM5	25	30	301	76
NSh-HZSM5	44	55	103	80
NS-HZSM5	23	33	218	103

^a^ Si/Al molar ratio calculated by XRF; ^b^ Si/Al molar ratio calculated by XRF and ^27^Al MAS NMR; ^c^ Concentration of pyridine adsorbed on Brønsted sites; ^d^ Concentration of pyridine adsorbed on Lewis sites.

**Table 3:** Thiele modulus for triglycerides transesterification using MC-HZSM5, NC-HZSM5, NSh-HZSM5, and NS-HZSM5.

	MC-HZSM5	NC-HZSM5	NSh-HZSM5	NS-HZSM5
ε(cm^3^·g^−1^)	0.165	0.166	0.323	0.624
$D_{eff}$(m^2^·s^−1^)	1.28 × 10^−7^	1.29 × 10^−7^	2.51 × 10^−7^	4.85 × 10^−7^
k(m^2^·h^−1^)	0.075	0.082	0.125	0.097
∅	0.1603	0.0050	0.0004	0.0033

## Data Availability

The data that supports the findings of this study are available within the article (and its Supplementary Materials).

## Supplementary Materials

Section S1: Zeolites’ gel compositions, Section S2: Catalysts Characterization, Section S3: Determination of the kinetics of the transesterification reaction, Section S4: 27Al MAS NMR spectra of (a) MC-HZSM-5, (b) NC-HZSM-5, (c) NSh-HZSM-5, and (d) NS-HZSM-5, Section S5: Yields of produced FAMEs and residual triglycerides and FFAs using MC-HZSM-5, NC-HZSM-5, NSh-HZSM-5 and NS-HZSM-5 zeolite catalysts, Section S5: Yields of produced FAMEs and residual triglycerides and FFAs using MC-HZSM-5, NC-HZSM-5, NSh-HZSM-5 and NS-HZSM-5 zeolite catalysts, Section S6: Wilke-Chang method used to calculate the molecular diffusion coefficient ($D_{AB}$), Section S7: -ln(1-YieldFAMEs) versus reaction time plot at optimal reaction conditions using MC-HZSM-5, NC-HZSM-5, NSh-HZSM-5 and NS-HZSM-5

## Sample Availability

Samples of the compounds are available from the authors.
