# Supplementary material for: Hierarchical Zeolites as Catalysts for Biodiesel Production from Waste Frying Oils to Overcome Mass Transfer Limitations
Source: Molecules. 2021 Aug 12;26(16):4879. doi: 10.3390/molecules26164879 (PMC8398946; doi:10.3390/molecules26164879)
Supplement: Supplementary file 1 [file molecules-26-04879-s001.zip › molecules-1294521-supplementary.pdf]

Supplementary Information

Hierarchical Zeolites as Catalysts for Biodiesel Production from Waste Frying Oils to Overcome Mass Transfer Limitations

Elyssa G. Fawaz [1], Darine A. Salam [1,*], Severinne S. Rigolet [2,3] and T. Jean Daou [2,3]

[1] Department of Civil and Environmental Engineering, Maroun Semaan Faculty of Engineering and Architecture, American University of Beirut, Riad El Solh, Beirut P.O. Box 11-0236, Lebanon; egf00@aub.edu.lb

[2] Axe Matériaux à Porosité Contrôlée (MPC), Institut de Science des Matériaux de Mulhouse (IS2M), Université de Haute Alsace, UMR CNRS 7361, ENSCMu, 68093 Mulhouse, France; severinne.rigolet@uha.fr (S.S.R.); jean.daou@uha.fr (T.J.D.)

[3] Université de Strasbourg, 67081 Strasbourg, France

* Correspondence: ds40@aub.edu.lb; Tel.: +961-(1)-350-000 (ext. 3609); Fax: +961-(1)-744-462

<u>Section S1:</u> <u>Zeolites' gel compositions</u>

HZSM-5 type	Gel composition
MC-HZSM-5	$100SiO_2$: $1Al_2O_3$: $30Na_2O$: $18H_2SO_4$: 20TPAOH: $4000H_2O$
NC-HZSM-5	$50SiO_2$: $1C_9H_{21}O_3Al$: 6NaBr: 10TPAOH: $450H_2O$
NSh-HZSM-5	$100SiO_2$: $1Al_2O_3$: $30Na_2O$: $18H_2SO_4$: $10C_{22}H_{45}$–$N^+(CH_3)_2$–C_6H_{12}– $N^+(CH_3)_2$–$C_6H_{13}Br_2$: $4000H_2O$
NS-HZSM-5	$100SiO_2$: $2.5Al_2O_3$: $22Na_2O$: 800EtOH: 5 $C_{18}H_{37}$–N^+ $(CH_3)_2$–C_6H_{12}–N^+ $(CH_3)_2$–C_6H_{12}– N^+ $(CH_3)_2$– $C_{18}H_{37}(Br^-)_3$: $7100H_2O$

Section S2: Catalysts Characterization**:**

X-ray diffraction patterns, purity and crystallinity of the produced zeolite materials were obtained using a PANalytical MPD X'Pert Pro diffractometer fitted with an X'Celerator real-time multi-strip detector (2.122 ° 2θ active length) and operating with Cu Kα radiation (λ = 0.15418 nm).

Morphology and homogeneity of the zeolites were studied by transmission electron microscopy (TEM) (Philips model CM200) and scanning electron microscopy (SEM) (Philips XL 30 FEG microscope).

Specific surface area (S_{BET}), micropore's size and volume (V_{micro}) of the produced zeolites were determined by N_2 sorption (ASAP 2420 system, Micrometitics, USA) and calculated adopting t-plot and BET processes. BJH method was used to calculate the mesoporous volume. Prior to single measurements, 50 mg of the zeolite samples were outgassed for 15 h, at 300 °C. Measurements of nitrogen sorption were carried out at -196 °C [58-59].

The Si/Al molar ratio of the synthesized zeolites was determined by X-ray fluorescence (Philips, Magic X).

The concentrations of Lewis and Brønsted acid sites were measured by pyridine adsorption trailed by infrared spectrometry in a Thermo Nicolet Magna 550-FT-IR spectrometer. Self-supported pellets of 20 mg of zeolite samples were squeezed at an equilibrium pressure of 1 Torr and preheated in an analysis cell at 450 °C for 12 h, in air. In order to remove physisorbed molecules, the cell was placed under vacuum for 1 h and then in the air to decrease the temperature to 200 °C. The temperature was further reduced to 150 °C before introducing pyridine into the cell for 5 min. The density of Lewis [PyrL] and Brønsted [$PyrH^+$] acid sites were established by integrating peaks' areas at 1545 cm^{-1} and 1454 cm^{-1} respectively using extinction coefficients previously determined by Guisnet et al. [60].

^{27}Al (I = 5/2) magic angle spinning (MAS) NMR was carried out with a Bruker Avance II 400 spectrometer operating at B_0 = 9.4 T (Larmor frequency ν_0 = 104.2 MHz) equipped with a Bruker 2.5 mm double channel probe. The rotor was spun at 25 kHz, and free induction decays (FID) were collected with a p/12 rf pulse (0.6 ms) and a recycle delay of 1 s. Measurements were carried out with $[Al(H_2O)_6]^{3+}$ as external standard reference [61]. The decompositions of the spectra were performed using the DMFit software [62] in order to determine the Si/Al ratio of the framework (coupled with XRF results).

To find the relative crystallinity of the different zeolites, the intensity of the first XRD peak of the microcrystals was considered as a reference, as it is a material with 100% relative crystallinity. The relative crystallinity of the other materials was then deduced correspondingly.

Section S3: Determination of the kinetics of the transesterification reaction

Transesterification reaction is divided into three steps whereby, triglycerides are converted to diglycerides, which then produce monoglycerides, which in turn produce glycerol.

Every step generates one ester molecule by consuming one mole of methanol and, consequently, the reaction produces three molecules of ester from one molecule of triglyceride [63]. The reaction will be assumed to be a single-step transesterification following the stochiometric relationship between the initial reactants and final products (eq. (2)):

$$Triglyceride + 3Methanol \xrightarrow{Catalyst} 3Fatty\ acid\ methyl\ esters\ +\ Glycerol \qquad 2$$

According to the general eq (2), the reaction rate can be expressed as follows (eq (3)):

$$-r_a = -\frac{1}{S}\frac{dC_{TGCs}}{dt} = k' \,.\, C_{TGCs} \,.\, C_{Me}^3 \qquad 3$$

Where r_a is the reaction rate (mg.L^{-1}.m^{-2}.h^{-1}), C_{TGCs} is the concentration of triglycerides (mg.L^{-1}), C_{Me} the concentration of methanol (mg.L^{-1}), S is the surface area of the solid catalyst, and k' the reaction rate constant (mg^{-1}.L.m^{-2}.h^{-1}).

The amount of triglycerides molecules adsorbed is equal to the sum of the amount desorped and the amount of triglycerides consumed by chemical reaction on the surface and intrisically [64]. According to the following assumptions, the reaction rate (1) is described as:

$$-r_a = -\frac{1}{S}\frac{dC_{TGCs}}{dt} = k'_s\theta_M C_{TGCs,s}.\, C_{Me,s}^3 \qquad 4$$

Where C_{TGCs} is the triglycerides concentration in the bulk liquid phase, k'_s is the chemical reaction constant on the catalyst surface, and θ_M is the occupied fraction of active sites by methanol, $C_{TGCs,s}$ is the concentration of triglycerides on the catalyst surface.
Corresponding to the suggested reaction mechanism, the adsorption of TGCs is the rate-limiting step. Then the net rate of TGCs adsorption-desorption is equal to the reaction rate on the catalyst surface (5):

$$r_{ads-des} = k_{ads}\,(1-\Sigma\theta)C_{TGCs} - k_{des}C_{TGCs,s} = -r_{LA} = -\frac{1}{S}\frac{dC_{TGCs}}{dt} = k_s C_{TGCs,s} \qquad 5$$

Where $r_{ads-des}$ is the net rate of TGCs adsortion-desorption, k_{ads} and k_{des} are the respective adsorption and desorption rate constants, $\Sigma\theta$ is the total solid fraction covered by all species in the liquid mixture.
Equation 5 can be reorganized to get the non measurable $C_{TGCs,s}$ in respect of measurable C_{TGCs} (6):

$$C_{TGCs,s} = \frac{k_{ads}\,(1-\Sigma\theta)}{k_{des} + k_s\theta_M} C_{TGCs} \qquad 6$$

Substitution of Equation (6) into Equation (4) results in Equation (7):

$$-r_a = -\frac{1}{S}\frac{dC_{TGCs}}{dt} = (k_s\theta_M)\frac{k_{ads}\,(1-\Sigma\theta)}{k_{des} + k_s\theta_M} C_{TGCs}C_{Me}^3 \qquad 7$$

All reactions can be viewed as the result of two consecutive steps. The first is the meeting of two reactant molecules by diffusion. The second is the reaction step, in which the reactants conquer

an activation barrier and a diffusion limitation. The transesterification reaction in the presence of a zeolite catalyst is reaction-controlled. As a result, the observed rate coefficient is only relevant to the reaction step and is equal to the intrinsic rate coefficient [65]. As discussed above, assuming that the adsorbed reaction components governed by the net rate of TGCs adsortion-desorption, contribute to the intrinsic reaction, the intrinsic rate coefficient (k_{eff}) would be (8):

$$k_{eff} = (k_s \theta_M) \frac{k_{ads}\,(1 - \Sigma\theta)}{k_{des} + k_s \theta_M} \qquad 8$$

Equation (4) thus becomes:

$$-r_a = -\frac{1}{S}\frac{dC_{TGCs}}{dt} = k_{eff} C_{TGCs} C_{Me}^3 \qquad 9$$

Where k_{eff} ($mg^{-1}.L.m^{-2}.h^{-1}$) reflects both the chemical reaction and adsorption-desorption resistance on the surface.

Eq. (9) follows a second order reaction rate. Nonetheless, the transesterification reaction is reversible and amounts of methanol in excess are needed to move the equilibrium to the side of the product [66]. Therefore, one could consider the variation in the concentration of methanol as constant during the transesterification reaction which will thus obey pseudo- first order kinetics [67-68]. The reaction rate can then be expressed as described in eq. (4):

$$-r_a = -\frac{1}{S}\frac{dC_{TGCs}}{dt} = k'.\,C_{TGCs}\,.\,C_{Me}^3 = k\,.\,C_{TGCs} \qquad 4$$

where *k* ($m^{-2}.h^{-1}$) = $k_{eff}.\,C_{ME}^3 \approx cst$, when methanol is used in excess.

Assuming that the initial concentration of triglycerides is $C_{0\,TGCs}$ at time *t* = 0 and becomes $C_{t\,TGCs}$ at time *t*, the integration of eq. (4) from *t* = 0 to *t* = *t*, and $C_{0\,TGCs}$ to $C_{t\,TGC}$ gives eq. (5):

$$ln\, C_{0\,TGCs} - ln\, C_{t\,TGCs} = k\,.\,t \qquad 5$$

From the mass balance of the reaction,

$$X_{FAMEs} = 1 - \frac{C_{TGCs}}{C_{0\,TGCs}}$$

where X_{FAMEs} is the fatty acid methyl ester yield. Upon rearrangement of eq. (5), the kinetics of triglycerides conversion could be expressed as follows (eq. (6)):

$$ln(1 - X_{FAMEs}) = -k\,.\,t \qquad 6$$

Section S4: ^{27}Al MAS NMR spectra of (a) MC-HZSM-5, (b) NC-HZSM-5, (c) NSh-HZSM-5, and (d) NS-HZSM-5. (* peak attributed to resonance band)

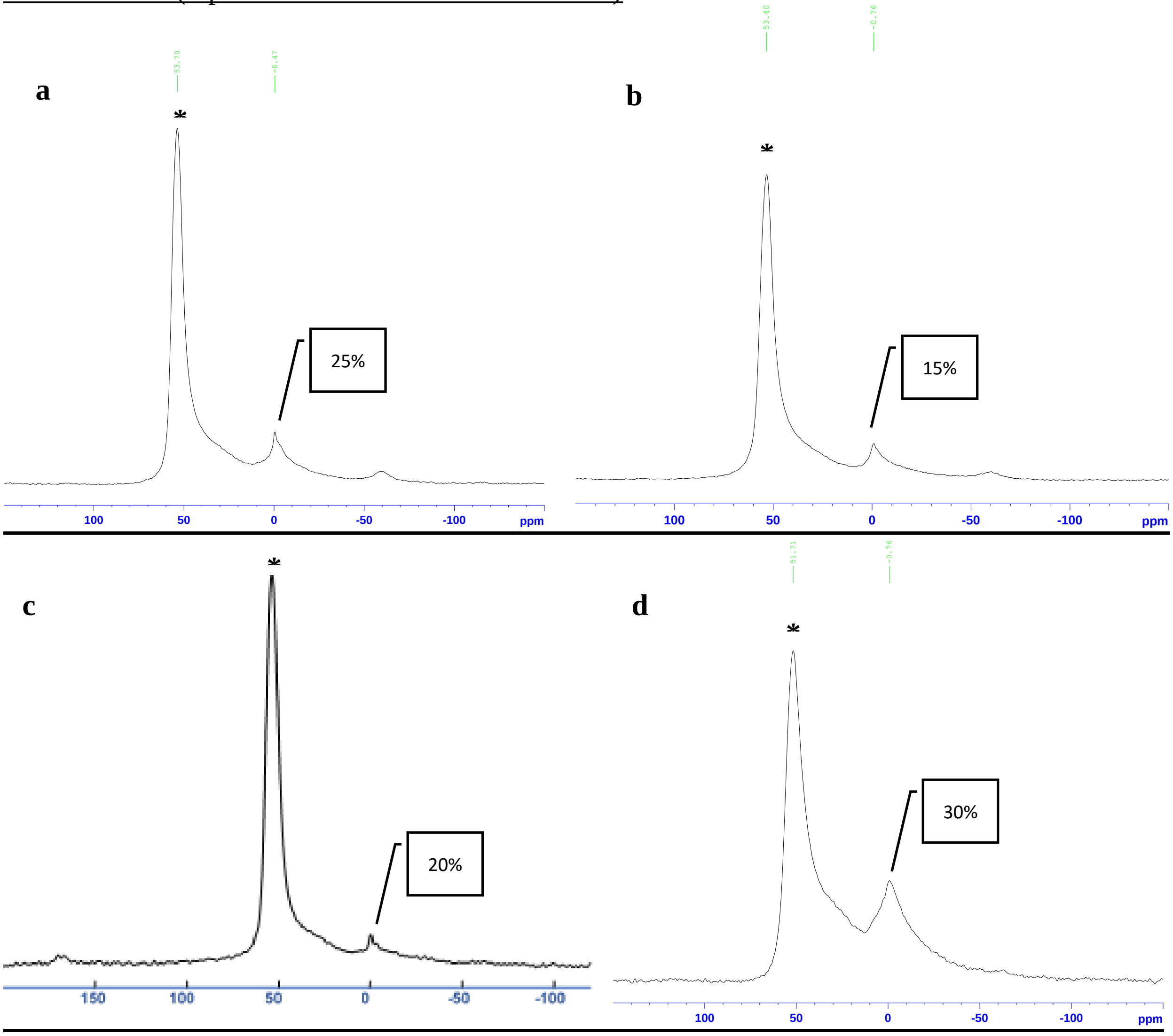

^{27}Al solid state MAS NMR spectra for the zeolite samples were recorded. The zeolites contained a principal resonance at ca. 56 ppm corresponding to Al species in tetrahedral coordination present in the MFI framework. ^{27}Al MAS NMR spectra also displayed a second resonance around 0 ppm attributed to extra framework Al. MC-HZSM-5, NC-HZSM-5, NSh-HZSM-5, and NS-HZSM-5, contain 25%, 15 %, 20 %, and 30 % of Al extra framework, respectively. Correspondingly, Si/Al framework ratios of the different zeolite samples were calculated.

Section S5: Yields of produced FAMEs and residual triglycerides and FFAs using MC-HZSM-5, NC-HZSM-5, NSh-HZSM-5 and NS-HZSM-5 zeolite catalysts

		MC-HZSM-5		NC-HZSM-5		NSh-HZSM-5		NS-HZSM-5	
Catalyst loading variation (wt%) at molar ratio of methanol to WFO of 12:1, reaction temperature of 140°C, and reaction time of 6 h		Average (%)	SD	Average (%)	SD	Average (%)	SD	Average (%)	SD
	FAMEs	6.73	1.72	8.00	2.25	19.90	0.31	12.18	2.57
5	Triglycerides	55.17	5.02	53.80	11.47	38.10	5.41	38.87	1.32
	FFAs	1.83	0.02	2.91	0.45	2.66	0.31	1.23	0.75
	FAMEs	14.97	3.10	15.34	2.66	24.62	0.42	16.94	1.29
7.5	Triglycerides	56.78	3.35	50.26	2.36	37.63	3.23	50.06	4.49
	FFAs	1.18	1.05	1.04	0.22	1.81	0.65	2.97	0.64
	FAMEs	15.60	0.57	17.29	0.14	26.84	1.59	19.43	0.18
10	Triglycerides	52.55	1.78	51.11	3.06	46.76	3.29	41.12	2.17
	FFAs	2.12	1.05	4.86	0.79	1.54	0.29	1.26	0.08
Methanol to LA ratio variation at 10% catalyst loading, reaction temperature of 140°C, and reaction time of 6 h									
	FAMEs	15.04	1.52	15.19	0.77	20.53	1.59	14.26	0.96
6 to 1	Triglycerides	34.06	17.72	44.36	6.42	50.37	0.89	47.49	1.19
	FFAs	1.71	0.63	1.07	0.83	4.17	2.79	2.31	0.36
	FAMEs	15.60	0.57	17.29	0.14	26.84	1.59	19.43	0.18
12 to 1	Triglycerides	52.55	1.78	51.11	3.06	46.76	3.29	41.12	2.17
	FFAs	2.12	1.05	4.86	0.79	1.54	0.29	1.26	0.08
	FAMEs	7.29	0.72	11.84	0.69	13.70	3.49	13.38	3.53
25 to 1	Triglycerides	64.96	0.23	52.41	1.74	55.45	2.44	61.87	2.58
	FFAs	1.91	0.28	2.11	0.34	1.50	0.81	1.37	0.08
Temperature variation (^{0}C), 10% catalyst loading, molar ratio of methanol to WFO of 12:1, and reaction time of 6 h									
	FAMEs					14.77	1.31		
60	Triglycerides					58.68	3.16		
	FFAs					1.63	0.49		
	FAMEs					26.84	1.59		
140	Triglycerides					45.26	2.39		
	FFAs					1.71	0.24		
	FAMEs					43.58	4.43		
180	Triglycerides					38.99	5.64		
	FFAs					1.02	0.26		

Reaction time variation (h) at 10% catalyst loading, molar ratio of methanol to WFO of 12:1, and reaction temperature of 140°C									
	FAMEs	7.07	0.04	6.13	0.79	4.50	0.04	6.02	2.59
0.5	Triglycerides	69.08	2.58	68.14	5.26	65.77	2.19	72.36	1.57
	FFAs	2.56	0.12	3.47	0.85	1.26	0.09	2.12	0.16
	FAMEs	9.47	0.34	11.44	0.08	6.65	2.05	8.99	1.84
1	Triglycerides	57.51	0.52	57.33	1.48	61.65	0.95	66.47	2.51
	FFAs	2.28	0.24	3.32	0.81	1.25	0.25	2.09	0.07
	FAMEs	14.68	0.12	15.43	1.11	13.68	2.74	13.15	0.49
2	Triglycerides	47.63	8.73	43.98	2.63	55.57	2.69	60.95	2.18
	FFAs	2.05	0.25	3.29	0.42	2.22	0.44	1.83	0.24
	FAMEs	19.91	0.15	18.49	0.76	20.45	0.10	22.08	3.71
3	Triglycerides	57.34	1.59	42.34	0.87	43.50	3.41	54.76	1.91
	FFAs	1.82	0.53	2.84	0.16	2.51	0.73	2.18	0.19
	FAMEs	23.46	0.53	26.40	1.03	46.11	2.19	33.20	1.06
4	Triglycerides	46.26	2.58	59.83	1.69	31.64	0.36	41.37	2.49
	FFAs	2.45	0.94	2.04	0.48	2.84	0.84	2.04	0.37
	FAMEs					43.58	4.43		
6	Triglycerides					26.02	2.73		
	FFAs					3.32	0.57		
	FAMEs					43.36	3.50		
8	Triglycerides					32.54	1.10		
	FFAs					1.64	0.40		
	FAMEs					31.21	3.58		
16	Triglycerides					37.64	5.77		
	FFAs					1.39	1.08		
	FAMEs					34.11	0.69		
24	Triglycerides					30.54	6.14		
	FFAs					1.49	0.19		

Section S6: Wilke-Chang method used to calculate the molecular diffusion coefficient (D_{AB})

$$D_{AB} = \frac{7.4\ .\ 10^{-8}(\partial\ M_B)^{1/2}\ T}{\varphi_B\ V_A^{0.6}}$$

Where,
M_B is the molecular weight of solvent B (methanol) (g/mol)
T is the temperature of the reaction (Kelvin)
φ_B is the viscosity of solvent B at T *(cP)*
V_A molar volume of solute A at T (m^2/mol)
∂ association factor of solvent B (dimentioneless; 1.9 for methanol)

Section S7: -ln(1-Yield$_{FAMEs}$) versus reaction time plot at optimal reaction conditions using MC-HZSM-5, NC-HZSM-5, NSh-HZSM-5 and NS-HZSM-5

To generate the reaction rates of the MC-HZSM-5, NC-HZSM-5 and NS-HZSM-5 zeolites, WFOs transesterification was carried out using catalyst loading of 10 wt%, methanol to linoleic molar ratio of 12:1, reaction temperature of 180 °C, stirring rate of 550 rpm, and with a reaction time range of 0 - 4 h.

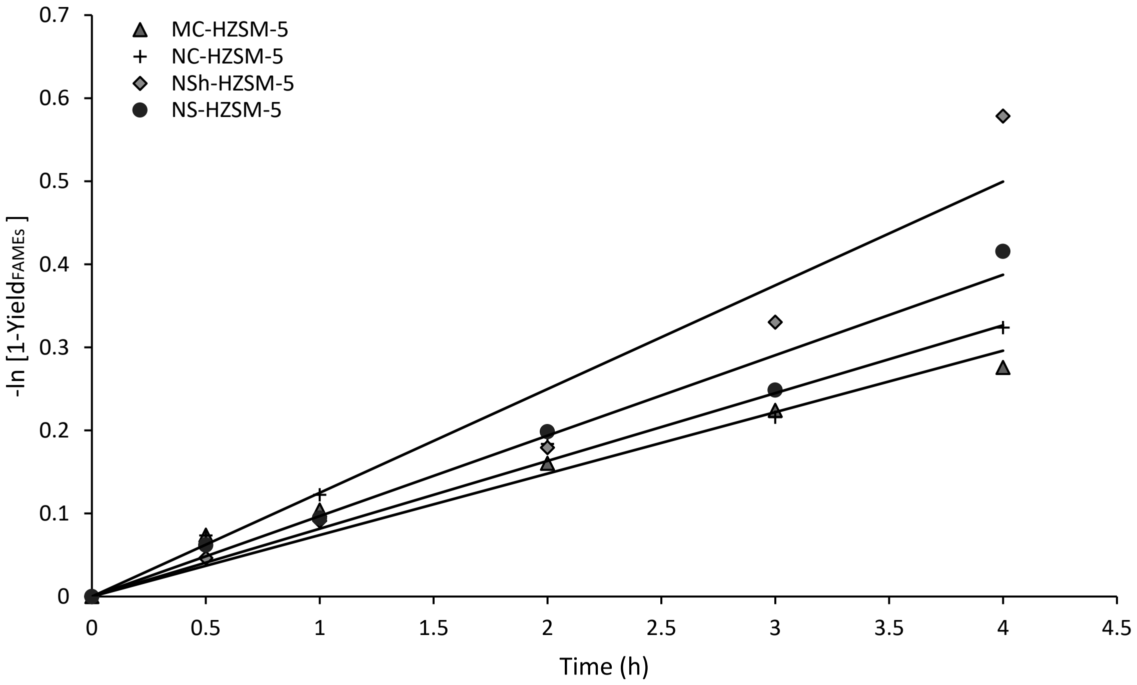

References

58. S. Brunauer, P.H. Emmett, E. Teller, Adsorption of Gases in Multimolecular Layers, J. Am. Chem. Soc. 60 (1938) 309–319. doi:10.1021/ja01269a023.
59. B. LIPPENS, Studies on pore systems in catalysts V. The t method, J. Catal. 4 (1965) 319–323. doi:10.1016/0021-9517(65)90307-6.
60. J. Guisnet, M., Ayrault, P., & Datka, Acid properties of dealuminated mordenites studied by IR spectroscopy. 2. Concentration, acid strength and heterogeneity of OH groups, Pol. J. Chem. 71 (1997) 1455–1461.
61. A. Astafan, Y. Pouilloux, J. Patarin, N. Bats, C. Bouchy, T. Jean Daou, L. Pinard, Impact of extreme downsizing of *BEA-type zeolite crystals on n-hexadecane hydroisomerization, New J. Chem. 40 (2016) 4335–4343. doi:10.1039/C5NJ02837J.
62. D. Massiot, F. Fayon, M. Capron, I. King, S. Le Calvé, B. Alonso, J.-O. Durand, B. Bujoli, Z. Gan, G. Hoatson, Modelling one- and two-dimensional solid-state NMR spectra, Magn. Reson. Chem. 40 (2002) 70–76. doi:10.1002/mrc.984.
63. Y.C. Sharma, B. Singh, Development of biodiesel from karanja, a tree found in rural India, Fuel. 87 (2008) 1740–1742. doi:10.1016/j.fuel.2007.08.001.
64. A.S. Abbas, T.M. Albayati, Z.T. Alismaeel, A.M. Doyle, Kinetics and Mass Transfer Study of Oleic Acid Esterification over Prepared Nanoporous HY zeolite, Iraqi J. Chem. Pet. Eng. 17 (2016) 47–60.
65. T. De Roo, J. Wieme, G.J. Heynderickx, G.B. Marin, Estimation of intrinsic rate coefficients in vinyl chloride suspension polymerization, Polymer (Guildf). 46 (2005) 8340–8354. doi:10.1016/j.polymer.2005.06.091.
66. Y. Chisti, Biodiesel from microalgae, Biotechnol. Adv. 25 (2007) 294–306. doi:10.1016/j.biotechadv.2007.02.001.
67. T.H. Dang, B.H. Chen, D.J. Lee, Application of kaolin-based catalysts in biodiesel production via transesterification of vegetable oils in excess methanol, Bioresour. Technol. 145 (2013) 175–181. doi:10.1016/j.biortech.2012.12.024.
68. A. Birla, B. Singh, S.N. Upadhyay, Y.C. Sharma, Kinetics studies of synthesis of biodiesel from waste frying oil using a heterogeneous catalyst derived from snail shell, Bioresour. Technol. 106 (2012) 95–100. doi:10.1016/j.biortech.2011.11.065.
